# Protocol for the Weight-bearing in Ankle Fractures (WAX) trial: a multicentre prospective non-inferiority trial of early versus delayed weight-bearing after operatively managed ankle fracture

**DOI:** 10.1186/s12891-021-04560-7

**Published:** 2021-08-09

**Authors:** C. P. Bretherton, H. A. Claireaux, J. Achten, A. Athwal, S. J. Dutton, N. Peckham, S. Petrou, R. S. Kearney, D. Appelbe, X. L. Griffin

**Affiliations:** 1 Nuffield Department of Orthopaedics, Rheumatology and Musculoskeletal Sciences, University of Oxford, Oxford, UK; 2 Nuffield Department of Primary Care, University of Oxford, Oxford, UK; 3 Department of Musculoskeletal Rehabilitation, University of Warwick, Coventry, UK; 4 Trauma and Orthopaedic Surgery, Division of Orthopaedics, Barts and The London School of Medicine and Dentistry, Queen Mary University of London, London, UK

**Keywords:** Ankle Fracture, Weight-bearing, Surgery, Rehabilitation, Randomised controlled trial

## Abstract

**Background:**

Unstable ankle fractures represent a substantial burden of disease, accounting for a mean hospital stay of nine days, a mean cost of £4,491 per patient and 20,000 operations per year. There is variation in UK practice around weight-bearing instructions after operatively managed ankle fracture. Early weight-bearing may reduce reliance on health services, time off work, and improve functional outcomes. However, concerns remain about the potential for complications such as implant failure. This is the protocol of a multicentre randomised non-inferiority clinical trial of weight-bearing following operatively treated ankle fracture.

**Methods:**

Adults aged 18 years and over who have been managed operatively for ankle fracture will be assessed for eligibility. Baseline function (Olerud and Molander Ankle Score [OMAS]), health-related quality of life (EQ-5D-5L), and complications will be collected after informed consent has been obtained. A randomisation sequence has been prepared by a trial statistician to allow for 1:1 allocation to receive either instruction to weight-bear as pain allows from the point of randomisation, two weeks after the time of surgery (‘*early weight-bearing*’ group) or to not weight-bear for a further four weeks (‘*delayed weight -bearing’ group*). All other treatment will be as per the guidance of the treating clinician.

Participants will be asked about their weight-bearing status weekly until four weeks post-randomisation. At four weeks post-randomisation complications will be collected. At six weeks, four months, and 12 months post-randomisation, the OMAS, EQ-5D-5L, complications, physiotherapy input, and resource use will be collected. The primary outcome measure is ankle function (OMAS) at four months post-randomisation.

A minimum of 436 participants will be recruited to obtain 80% power to detect a non-inferiority margin of -6 points on the OMAS 4 months post-randomisation. A within-trial health economic evaluation will be conducted to estimate the cost-effectiveness of the treatment options.

**Discussion:**

The results of this study will inform national guidance with regards to the most clinically and cost-effective strategy for weight-bearing after surgery for unstable ankle fractures.

**Trial registration:**

ISRCTN12883981, Registered 02 December 2019.

**Supplementary Information:**

The online version contains supplementary material available at 10.1186/s12891-021-04560-7.

## Background

One hundred seventy patients sustain an ankle fracture in the UK every day [1]. Ankle fractures are grouped into those in which the bones remain aligned (stable) and those in which they do not (unstable). Unstable fractures require surgery to stabilise the ankle bones whilst they heal [2]. 20,000 ankle operations are performed every year in the UK [3]. They represent a substantial burden of disease, with an average length of hospital stay of nine days and an associated mean cost of £4,491 per patient [4].

Post-operative mobilisation strategies are variable. Historically surgeons restricted patients’ weight-bearing due to a fear that excessive loading of the bone and metal implants could lead to an early loss of alignment, soft tissue compromise, poor functional outcomes and a requirement for revision surgery [5]. This weight-bearing restriction has a major impact on patients and health services through enforced use of walking aids, increased dependency and/or longer hospital stays [6–8]. Patients also report significant time off work with the consequent personal and societal cost impact [6, 8, 9]. It is reasonable that if there were minimal risk of harm, many patients would prefer to walk without weight-bearing restrictions.

High-quality systematic reviews conducted by Cochrane and the National Institute for Health and Care Excellence (NICE) have found no credible evidence of harm associated with an early weight-bearing strategy [2, 10]. There is a pressing need to definitively test the appropriateness of this component of the surgical treatment of ankle fractures.

## Methods

### Aims

This study aims to establish whether early weight-bearing after an operatively managed ankle fracture is not inferior in terms of functional outcome compared to delayed weight-bearing.

The primary outcome is:

1. To quantify and draw inferences on observed differences in ankle function between early and delayed weight-bearing using the Olerud and Molander Ankle Score (OMAS) at four months after randomisation.

The secondary outcomes are:

1. To quantify and draw inferences on observed differences in ankle function between early and delayed weight-bearing using the Olerud and Molander Ankle Score (OMAS) at six weeks and 12 months after randomisation.
2. To quantify and draw inferences on observed differences in health-related quality of life using the EQ-5D-5L between the trial treatment groups at six weeks, four months, and 12 months after randomisation.
3. To investigate the difference in risk of complications between the trial treatment groups at four weeks, six weeks, four months, and 12 months after randomisation.
4. To investigate the resource use, costs and comparative cost-effectiveness of the treatment options using the Work Productivity and Activity Impairment: Specific Health Problem (WPAI), prospectively measured resource use, and health utility estimates at six weeks, four months, and 12 months.

### Study design

This trial is a pragmatic, multicentre, randomised non-inferiority clinical trial with a parallel economic evaluation conducted in the United Kingdom. This protocol adheres to the Standard Protocol Items: Recommendations for Interventional Trials (SPIRIT) guidelines for reporting of clinical trial protocols (Additional file 1).

### Trial registration

ISRCTN12883981, Registered 02 December 2019, https://www.isrctn.com/ISRCTN12883981

### Protocol version

This publication is based on the WAX trial protocol version 3.0, approved by South Central- Oxford A- Health Research Authority on 29 March 2021.

### Eligibility

Patients will be eligible for inclusion into the trial if:

- They are aged 18 years and older.
- They have undergone operative fixation for an unstable ankle fracture.
- Surgery was performed within 14 days of the injury.
- In the opinion of the treating surgeon, the patient might benefit from early weight-bearing.
- They are able and willing to give informed consent.

Patients will be excluded from this trial if any of the following apply:

- They have a lack of protective sensation (e.g. peripheral neuropathy).
- They are unable to adhere to trial procedures.
- They have bilateral operatively treated ankle fractures.
- They are already in a trial for ankle fracture.
- They received a hindfoot nail to treat index fracture.

### Consenting

Patients will be approached by a member of the local recruitment centre team within two weeks of their surgery, to introduce them to the trial. Written and verbal versions of the Participant Information and Informed Consent documentation will be presented to the participants to explain the exact nature of the study.

### Randomisation

Those patients who consent to take part in the trial will be randomly allocated to one of two intervention groups (1:1) using a secure (encrypted) remote computer randomisation service administered by the Oxford Clinical Trials Research Unit (OCTRU).

Randomisation allocation will be implemented using a minimisation algorithm stratified by age and centre; the sequence will be prepared by the trial statistician.

#### Stratification by age

There is a bimodal incidence in ankle fractures, with the older population typically suffering low energy fragility fracture, whilst in the younger population, the injuries are more often the result of sports-related injuries. To align with recently published and ongoing NIHR multicentre randomised controlled trials (RCTs) on ankle fracture management, we have chosen to separate patients younger and older than 60 years old as the stratification cut-off to ensure balance across the treatment arms and for subgroup analysis for this trial [11, 12].

#### Stratification by centre

Stratification by centre will help ensure that any effect related to the recruitment centre will be equally distributed in the trial arms. All hospitals throughout the NHS are familiar with giving rehabilitation advice that patients should weight-bear or avoid weight-bearing after various injuries.

#### Post-randomisation withdrawals

A participant may choose to withdraw from the study at any time, without giving reasons, and without affecting their clinical care. Participants will not have the option to withdraw the data collected up until the point of withdrawal, as the data will be required for the intention-to-treat and safety analysis. Withdrawn patients or patients deemed ineligible after consent will not be replaced.

### Blinding

It will not be possible to blind participants or those delivering the intervention to the weight-bearing instruction. The primary outcome data will be collected from participants and entered directly onto the central trial database, without influence from the clinical or research team.

### Trial treatments

**Fig. 1:**
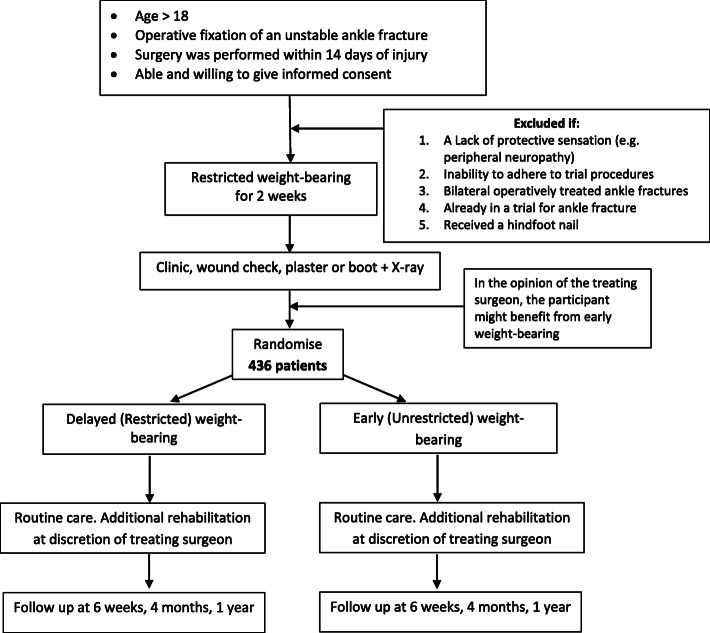
Patient Pathway

A patient pathway is included in Fig. 1.

### Pre-randomisation

All NHS hospitals are familiar with giving rehabilitation instructions for weight-bearing or non-weight-bearing. All patients will be non-weight-bearing for the first two weeks in the immediate post-operative period to allow swelling to settle and the wound to start healing. The type of ankle immobilisation will be decided as per usual care by the treating clinical team.

The timeframes and definitions of early and delayed weight-bearing are in line with NICE NG38 standards [10]. Clear instructions for patients to avoid weight-bearing if they experience pain or discomfort will act as an additional safety measure. The decisions for both groups of the trial about which form of immobilisation, such as a plaster, boot or other (if any) is used and how much unloaded ankle exercise is allowed will be at the treating clinician’s discretion but will be made prior to randomisation and will reflect their routine practice. This is a pragmatic trial so these co-interventions will not be specified; details of any will, however, be recorded on the Case Report Forms (CRFs). At their routine two-week post-surgery clinic visit, participants will be randomised to either early or delayed weight-bearing.

Subsequent formal rehabilitation or adjunctive therapies will be left to the discretion of the treating clinician (e.g. referrals to physiotherapy). The type, frequency and duration of these will be recorded as part of the six weeks, four months and 12 months post-randomisation follow-ups.

### Intervention: Early Weight-Bearing (unrestricted weight-bearing)

The local clinical and research teams will be trained in providing standardised weight-bearing instructions to participants; this will include a written training guide with key points to reinforce. Participants will receive the instructions for weight-bearing in verbal and written form. They will be instructed to put as much weight through their affected leg as they feel comfortable and without causing pain. They will be informed that while they are allowed to walk on their operated leg, it does not mean they have to, or that they will feel able to immediately. They can continue to use the walking aids provided to them during their inpatient hospital stay, or they can discard them if they feel confident to walk without them at any point during the following four weeks.

### Comparator: Delayed Weight-Bearing (restricted weight-bearing)

A standardised verbal and written instruction to continue to delay weight-bearing for a further four weeks from the point of being randomised will be given to the patient. They will be instructed to avoid putting any weight through their operated leg. They will be asked to continue to use the walking aids provided to them during their inpatient hospital stay.

### Rehabilitation adherence and fidelity

Training of rehabilitation providers will be undertaken at each site by the WAX research team. The rehabilitation materials will be delivered using standardised verbal, written and/or electronic/online instructions. Adhering to the Template for Intervention Description and Replication (TIDIeR) checklist for description and replication of rehabilitation interventions [13], the initial rehabilitation instructions will be recorded on a rehabilitation CRF. In addition, the interactions between the clinician and trial participant will be recorded (verbal consent will be sought) and evaluated for at least one participant during the recruitment and intervention delivery phase of the trial at each recruitment centre. These recordings will be used to assess the level of success of the clinician to introduce the aims/rationale of either intervention strategy.

Objective measurement of weight-bearing through direct pedal pressure monitoring or gait assessment was deemed infeasible. Adherence assessment within this study will be based upon participant self-report. Participants in both groups will receive weekly emails/SMS messages (a total of 4) asking them to confirm if they have started weight-bearing.

### Adverse events management

Safety reporting for each participant will begin at randomisation and will end when the participant has reached their final main follow up time point, at 12 months post-randomisation. When the local research team becomes aware of a Serious Adverse Event (SAE) in a trial participant, the Principal Investigator (PI) will review the SAE locally and make a decision about the causality. All expected and related events shall be reported as complications rather than SAEs. For any SAEs assessed as unexpected and potentially related, the details of the event will be entered on an SAE reporting form on the database, and the local research team will notify the central team via email or telephone within 24 hours of the PI becoming aware of the event. Once the SAE form is received, causality will be confirmed by the Chief Investigator (CI) or delegate (Nominated Person). In the event that consensus is not reached between the PI and Nominated Person about assessment of causality and expectedness, this will be escalated to the CI for further discussion. All such events will be reported to the Trial Steering Committee (TSC) and Data and Safety Monitoring Committee (DSMC) at their next meetings and to the Research Ethics Committee (REC) that issued favourable opinion within 15 working days. All participants experiencing SAEs will be followed up as per protocol until the end of the trial. As both groups are investigating procedures currently used in clinical practice, we will not be collecting unrelated SAEs.

### Outcome measurements

The primary outcome measure is the Olerud and Molander Ankle Score (OMAS) [14]. It is a reliable and valid ankle-specific patient-reported outcome measure used in several other ankle fracture studies [2, 11, 12, 15]. It has three domains related to ankle symptoms, lower limb-related activities, and overall function. It has nine items that add to give a score of 0 (worst) to 100 (best). A difference of five points would represent someone who is able to walk upstairs without any difficulty compared to someone who could manage stairs, but with some difficulty.

The instrument will be collected at baseline (patients will be asked to recall their pre-injury ankle function), six weeks, four months and 12 months post-randomisation.

The secondary outcome measures are:

#### Health-related quality-of-life

The EuroQol 5 Dimensions 5L (EQ-5D-5L) [16] is a validated, generalised and standardised instrument comprising a visual analogue scale measuring self-rated health and a health status descriptive system. For analysis, responses to the descriptive system will be converted into EQ-5D-3L health utility scores using a published utility algorithm for the UK population, as recommended by NICE [17]. For the baseline measurement, patients will be asked to recall their pre-injury health state.

#### Return to work

The Work Productivity and Activity Impairment: Specific Health Problem (WPAI) is a validated instrument [18] that measures the impact of health and symptom severity on work productivity and non-work activities.

#### Complications

All potentially related complications will be recorded, including fracture displacement, non-union, malunion, infection, symptomatic deep vein thrombosis, pulmonary embolus, and unplanned return to theatre.

#### Resource use

NHS, social care, and personal out-of-pocket costs, including a comparison of return-to-work time between groups.

#### Radiological outcome

Routinely collected radiographs will be harvested. An independent adjudication committee will assess the radiographs for intra-operative mal-reduction and postoperative loss of reduction. Loss of reduction will be defined as a radiograph demonstrating any one or combination of showing talar subluxation > 2 mm (talar shift), excessive talar tilt (> 2°) or a diastasis (tibiofibular clear space ≥ 5 mm) [19].

A schedule of study procedures is included in Table 1.

**Table 1:** Schedule of study procedures

	Screening	Enrolment	4 weeks	6 weeks	4 months	12 months
	-14 days	Day 0	(± 2 weeks)	(+- 2 weeks)	(±8 weeks)	(±8 weeks)
**Enrolment**						
Eligibility assessment	X	X				
Informed consent		X				
Allocation		X				
**Interventions**						
Early weight bearing		X				
Delayed weight bearing		X				
**Baseline characteristics**						
Demographics		X				
Educational attainment		X				
Work status		X				
Tampa scale for Kinesphobia		X				
Self-efficacy report		X				
Modified Charlson Comorbidity Index		X				
Index of Multiple Deprivations		X				
Residential status		X				
Discharge destination		X				
**Assessments**						
OMAS / EQ-5D-5L		X		X	X	X
Complications		X	X	X	X	X
Weight-bearing diary			X			
Physiotherapy input				X	X	X
Resource use				X	X	X
Qualitative interviews				X		

We will use established techniques to ensure minimum loss to follow-up, including collecting multiple contact addresses, telephone numbers, and email addresses.

### Sample size

The primary clinical outcome is OMAS at four months post-randomisation [14], as function has been shown to improve quickly in the first three months from surgery, improve little between three and six months and plateau thereafter [20]. Previous studies have demonstrated a minimally clinical important difference of 10 points for this instrument [11, 14] which is in accordance with expert opinion (for scales scoring 0-100) and statistical convention [21]. We have selected a standard deviation (SD) of 21.1 based on the largest published RCT reporting OMAS within six months for operatively treated ankle fractures [11]. Assuming a baseline SD (pre-injury) of 21.1 and a non-inferiority margin of -6 points on the OMAS, based upon data from the AIM trial, [11] 392 participants providing data at four months (196 per arm) will provide 80% power and 2.5% (1-sided) significance. Allowing for 10% loss to follow-up (4.4% at six months in the AIM trial) [11] this yields an overall minimum target of 436 participants (218 per arm). If early weight-bearing is found to be non-inferior to delayed weight-bearing then superiority will also be tested at 2.5% (1-sided) significance.

### Study within a trial

A Study Within a Trial (SWAT) will investigate the generalisability, acceptability and mechanism of action of the trial interventions using CRFs, enhanced screening Logs, and patient and staff interviews at six weeks post-randomisation. Recruitment for the semi-structured interviews and discussion workshops will occur alongside the initial WAX trial consent discussion. The SWAT will be published separately, and a protocol is available in the supplementary materials (Additional file 2).

### Analysis

#### Statistical analysis

A separate Statistical Analysis Plan (SAP) complete with full details of the statistical analyses planned for this study will be drafted and finalised prior to the primary outcome analysis.

Non-inferiority trials assess whether an intervention is not clinically worse than a control, and therefore the interest is one-sided. The OMAS score at four months post-randomisation is the primary outcome in this study and we have defined a non-inferiority margin (ΔT) of -6 points, which is the maximum difference we are prepared to tolerate and still consider early weight-bearing not to be clinically inferior to delayed weight-bearing. The null hypothesis is therefore that a difference of greater than ΔT exists in favour of delayed weight-bearing (H0: Δ ≤ -ΔT). This will be assessed by creating a 95% confidence interval (95% CI), which should be entirely above the non-inferiority margin for the intervention to be declared non-inferior. As well as assessing if non-inferiority (and superiority) is demonstrated, sensitivity analyses will be undertaken to assess a range of potential biases that could have resulted from loss-to-follow-up, protocol deviations, withdrawal (including mortality).

The result of the analysis for the primary endpoint should be one of the following:

- The 95% CI lies entirely above the non-inferiority margin (-Δ_T_), so that non-inferiority may be concluded with only a small probability of error.
- The 95% CI includes points below the non-inferiority margin, then there is a possibility that the intervention is inferior to the control and non-inferiority cannot be safely concluded.
- The 95% CI is entirely above zero, indicative of a treatment effect, then superiority of the intervention can be concluded within a small probability of error.
- The 95% CI is entirely below the non-inferiority margin, indicative that the intervention is clinically inferior to the control.

All analyses will be carried out on the intention-to-treat (ITT) population and repeated for the per protocol population. Non-inferiority will be concluded only if both analyses show non-inferiority. This is due to the potential conservative nature of applying the ITT analysis only in a non-inferiority trial. A per-protocol analysis could introduce bias into the treatment comparison if there are differential numbers of protocol deviations in the different arms. Therefore, we will also undertake a Complier Average Causal Effect analysis which essentially compares the “compliers” in each group and is therefore randomisation preserving. This is therefore less prone to bias than a per-protocol analysis, although if the number of non-compliers is high this may affect the interpretation as the power is reduced for smaller subgroups (the compliers).

The OMAS score will be compared between treatment groups as the dependent variable in a mixed-effects linear regression model for the primary analysis with adjustments for stratification factors and baseline (pre-injury) OMAS score. A random effect will be included to account for any heterogeneity in the response due to recruitment centre. Fixed effects will be included to adjust for participant age and gender. The treatment difference will be based on the estimate of adjusted means and 95% confidence intervals.

Secondary clinical outcomes will be analysed using mixed effects regression, using logistic regression for binary data and linear regression for continuous data. Supplementary analyses to explore the recovery trajectory between the two treatments will be conducted for the OMAS score using the area under the curve summary statistics [14, 22].

### Health economics evaluation

A prospective economic evaluation, conducted from an NHS and personal social services perspective, will be integrated into the trial design. The economic evaluation will estimate the difference in the cost of resource inputs used by participants in the two arms of the trial, allowing comparisons to be made between the two weight-bearing strategies following ankle fracture fixation and enabling costs and consequences to be compared. The economic assessment method will, as far as possible, adhere to the recommendations of the National Institute for Health and Care Excellence (NICE) Reference Case.

Primary research methods will be followed to estimate the costs of the treatment options, including supplementary interventions (e.g. revision surgery) and rehabilitation inputs. Broader resource utilisation will be captured through to principal sources: (1) routine health service data collection systems and (2) patient questionnaires administered at baseline, six weeks and four and 12 months post-randomisation. Unit costs for health and social care resources will largely be derived from local and national sources and estimated in line with best practice. Primary research using established accounting methods may also be required to estimate unit costs. Costs will be standardised to current prices where possible. Health-related quality of life will be measured at baseline preinjury, six weeks, four and 12 months post randomisation using the EuroQol EQ-5D-5L measure; responses will be used to generate quality-adjusted life-years (QALYs). Utility weights will be estimated using recommended algorithms until a national tariff set for the EQ-5D-5L is available. We will in the first instance use self-reports of the EQ-5D measure. Where these data are not available, we will estimate health utilities at each time point using mapping equations between the OMAS and EQ-5D health outcomes on the basis of existing datasets held by the trial team.

Multiple imputation methods will be used to impute missing data and avoid biases associated with complete case analysis. The results of the economic evaluation will be expressed in terms of incremental cost per QALY gained. We shall use non-parametric bootstrap estimation to derive 95% CIs for mean cost differences between the trial groups and to calculate 95% CIs for incremental cost-effectiveness ratios. A series of sensitivity analyses will be undertaken to explore the implications of uncertainty on the incremental cost-effectiveness ratios and to consider the broader issue of the generalisability of the study results. One such sensitivity analysis will involve adopting a societal perspective for the economic evaluation, which will incorporate direct costs to trial participants, informal care provided by family and friends and productivity losses. In the baseline analysis, and for each sensitivity analysis, cost-effectiveness acceptability curves will be constructed using the net-benefits approach. Heterogeneity in the trial population will be explored by formulating net-benefit values for trial participants from the observed costs and effects and then constructing a regression model with an intervention variable and covariates such as age and sex. The magnitude and significance of the coefficients on the interactions between the covariates and the intervention variable will provide estimates of cost-effectiveness of the treatment options by participant subgroup.

### Trial oversight

A Trial Steering Committee (TSC) and Data and Safety Monitoring Committee (DSMC) will be set up. The study DSMC will adopt a DAMOCLES charter which defines its terms of reference and operation in relation to oversight of the trial. They will not be asked to perform any formal interim analyses of effectiveness. They will, however, review accruing data, summaries of the data presented by treatment group, and will assess the screening algorithm against the eligibility criteria. They will also consider emerging evidence from other related trials or research and review related SAEs that have been reported.

This study will be conducted as part of the portfolio of trials in the registered UKCRC Oxford Clinical Trials Research Unit (OCTRU) at the University of Oxford. It will follow their Standard Operating Procedures ensuring compliance with the principles of Good Clinical Practice and the Declaration of Helsinki and any applicable regulatory requirements. The OCTRU Quality Assurance team will audit the trial at least once in the lifetime of the study, more if deemed necessary.

### Dissemination

A manuscript will be submitted to a high impact peer-reviewed journal. Authorship will be determined in accordance with the International Committee of Medical Journal Editors guidelines [23] and other contributors will be acknowledged.

## Discussion

The results of this study will inform national guidance with regards to the most clinically and cost-effective strategy for weight-bearing after surgery for unstable ankle fractures.

### Sponsor

University of Oxford, Joint Research Office, 1st floor, Boundary Brook House Churchill Drive, Headington, OX3 7GB. ctrg@admin.ox.ac.uk. The Sponsor reviews the trial protocol and amendments before submission to REC but delegates the study design, management, analysis, interpretation of the data, report writing and decision to submit for publication to the CI and OCTRU.

## Supplementary Information

**Additional file 1.** The SPIRIT checklist with page numbers.

**Additional file 2.** The WAX SWAT study protocol.

## Data Availability

Not applicable.

## Abbreviations

AE: Adverse Event
CI: Chief Investigator
CRF: Case Report Form
DSMC: Data and Safety Monitoring Committee
EQ-5D-5L: EuroQol 5 Dimensions 5L
NICE: National Institute for Health and Care Excellence
OCTRU: Oxford Clinical Trials Research Unit
OMAS: Olerud and Molander ankle score
PI: Principal Investigator
QALY: Quality Adjusted Life Year
RCT: Randomised Controlled Trial
SAE: Serious Adverse Event
SAP: Statistical Analysis Plan
SD: Standard Deviation
SWAT: Study Within a Trial
TIDiER: Template for Intervention Description and Replication
TSC: Trial Steering Committee
WAX: Weight-Bearing in Ankle Fractures
WPAI: The Work Productivity and Activity Impairment: Specific Health Problem
