# Supplementary material for: Protocol for the Weight-bearing in Ankle Fractures (WAX) trial: a multicentre prospective non-inferiority trial of early versus delayed weight-bearing after operatively managed ankle fracture
Source: BMC Musculoskelet Disord. 2021 Aug 9;22:672. doi: 10.1186/s12891-021-04560-7 (PMC8353856; doi:10.1186/s12891-021-04560-7)
Supplement: Supplementary file 2 — Additional file 2. The WAX SWAT study protocol. [file 12891_2021_4560_MOESM2_ESM.docx]

**WAX Study Within A Trial (SWAT) Protocol:**

**Rehabilitation After Ankle Fracture Treatment (RAAFT): Facilitating safe weight-bearing after ankle fracture surgery**

CP Bretherton, H Sandhu, J Baird, XL Griffin

**INTRODUCTION**

The Weight-bearing in Ankle Fractures (WAX) trial is an NIHR Research for Patient Benefit (RfPB) study that will randomise patients to weight-bear early (at two-weeks postoperatively) or wait till six weeks postoperatively, which is the current standard of care.^1,2^ It will also conduct health economic analysis to determine the cost-effectiveness of the two weight-bearing strategies.

Recent qualitative studies have highlighted the burden for patients having to endure a period of non-weight bearing.^3,4^ While they often report difficulty with non-weight-bearing, it is not known how patients respond once permitted to weight-bear.  Even if instructed to weight-bear, many patients are wary of pain, falls and reinjury and so avoid walking on their ankle for several weeks.^4^

We do not know how patients decide what is an acceptable level of discomfort when starting to weight-bear (whether this is undertaken early or late) or how they decide to stop using their post-operative splints or walking aids.  There is little guidance for clinicians on advising patients and no rehabilitation package for patients themselves.

An intervention to encourage weight-bearing would be considered a “complex intervention”.^5^ This is because techniques to change weight-bearing behaviour would include several interacting components, which may be related to those delivering (healthcare professionals) and those receiving the intervention (patients).^5^ Behaviour change theory has been used to design and evaluate similarly complex rehabilitation interventions in chronic musculoskeletal conditions.^6^ This framework allows the linkage of determinants of behaviour (e.g. pain beliefs or resource availability) to behaviour change techniques, which can be used to develop an intervention.^7^

**We therefore propose:**

Co-production of a rehabilitation package to facilitate safe weight-bearing after ankle fracture surgery based on the behaviour change wheel.

# STUDY DESIGN

This Study Within a Trial (SWAT) has three work packages:

1. An embedded exploratory study investigating the impact of adverse pain beliefs in patients with surgically managed ankle fracture.
2. Conduct an analysis of interviews of patients and healthcare professionals participating in the WAX trial. This analysis will investigate patients’ experience of pain, recovery and commencing weight-bearing.
3. Co-design a rehabilitation package to facilitate weight-bearing after ankle fracture surgery, based on the behaviour change wheel. This will be through a series of workshops with healthcare professionals and patients.

# METHODOLOGY

## Study Participants

- Patients having undergone operative fixation of an unstable ankle fracture that have been approached to take part in the WAX trial
- Healthcare professionals that have been involved in the WAX trial.

## Recruitment

Recruitment will be embedded in the WAX trial recruitment process.

Separate consent forms allow patients who decline participation in the WAX trial to be invited for interviews and workshops to ensure the qualitative study is maximally representative of an ankle fracture population

Additionally, patients, carers and healthcare professionals will be recruited through professional and charitable societies including Arbeitsgemeinschaft für Osteosynthesefragen (AO)UK, the Orthopaedic Trauma Society and through local Patient and Public Involvement (PPI) networks and those of existing patient representatives.

## Consent

As part of the initial consent discussion for the WAX trial, patients will be asked whether they may be approached about participating in interviews and workshops. Prior to interview, participants will receive written and verbal information about participating in an interview. Participants who agree to be approached will have a separate informed consent discussion for the interviews with a member of the research team either in person, or by phone. NHS staff will initially be invited to interview by phone or email, and if they agree to be approached, an informed consent discussion will take place with a researcher from the WAX office by phone, videocall or in person.

# SWAT PROCEDURES

The study will follow the Medical Research Council framework for developing and evaluating complex interventions^5^:

- Identify the evidence base (Completed pre-RAAFT)
- Identify/ develop theory (work package 1 and 2)
- Model process/ outcomes (work package 3)

**Work package 1: An embedded exploratory study investigating the impact of adverse pain beliefs**

Baseline characteristics and variables for linear regression models:

Additional patient characteristics are embedded into the baseline demographic data collection in the WAX trial. They include:

- Educational attainment
- The Charlson comorbidity index
- The Index of Multiple Deprivations (IMD)

Two short questionnaires will explore participants’ pain beliefs:

**Fear of movement**: The Tampa Scale for Kinesiophobia -11 is a brief, reliable, and valid measure of fear of movement/(re)injury for patients with chronic painful conditions.^8^

**Pain self-efficacy**: The pain self-efficacy questionnaire-2 is a validated, abbreviated measure of an individual’s confidence in their ability to accomplish tasks, overcome problems and live a normal life despite pain.^9^

The other predictor variables already collected as part of the WAX trial include age, gender, occupation (sedentary vs manual labour), injury mechanism and blinded weight-bearing allocation.

Outcome Measurements

The primary outcome measure for the exploratory analysis is the Olerud and Molander Ankle Score (OMAS) measured at 4 months post-randomisation. It is a reliable and valid ankle-specific patient-reported outcome measure used in several other ankle fracture studies. ^10–12,13^.

The secondary outcome measures are Health-related quality-of-life (EQ-5D-5L) and Adverse events (AEs).

Sample size/ available data:

The sample size for the WAX trial is 436 patients, with a predicted 10% loss to follow-up rate. With 10 predictor variables, 80% power and a significance level of 0.05, the regression model would be able to detect and account for 4% of the variability in OMAS at 4 months (F^2^=0.042 = R^2^=0.040; calculations performed using G*Power3.1.9.4). This R^2^ is much lower than the expected adjusted R^2^ based on previous studies.^14,15^

## Work package 2: Qualitative interviews

### Semi-structured interviews with patients

Participants’ experiences of treatment and barriers and facilitators to commencing weight-bearing after ankle fracture surgery will be explored. Participants will be invited to take part in an interview at around 8 weeks after their surgery. Interviews will be up to 60 minutes and conducted by telephone or videocall. All interviews will be audio-recorded, anonymised and transcribed verbatim. Interviews will be semi-structured and use a brief, flexible topic guide.

Maximum variation sampling strategy to ensure the spread of patient characteristics by age (age over or under 60), and pain self-efficacy (scores higher or lower than eight based on mean results from previous studies^14,15^). Participant interviews will continue until data saturation is achieved. Based on a previous study, it is estimated data saturation will be achieved in around 25-30 interviews. ^4^

### Semi-structured Interviews with surgeons and healthcare professionals

25-30 Healthcare professionals, including surgeons, physiotherapists and research nurses will be sampled to reflect the multidisciplinary care team delivering care and research interventions to patients after ankle fracture. They will be invited to an interview to discuss their perceptions of how patients make sense of current weight-bearing instructions and any common difficulties they express.

##

**Work package 3: Iterative co-design of a rehabilitation package to facilitate safe weight-bearing**

Informed by work package 1 and 2, a rehabilitation strategy will be co-designed, using the three phases of the Behaviour Change Wheel (BCW). This technique is informed by extensive theory and has been used to design and implement rehabilitation interventions.^7^ The three phases are:

1. *Understand the behaviour* by assessing participants’ capability, opportunity and motivation (COM-B) to engage with the behaviour (weight-bearing). These will be derived from the qualitative interviews. These will then be mapped to the 9 intervention functions of the Behaviour Change Wheel.^7^ A set of candidate behavioural change intervention bundles will then be generated.
2. *Understand intervention options* using the APEASE (affordability, practicability, effectiveness/ cost-effectiveness, acceptability, safety/ side effects and equity) criteria.^16^

Workshop 1. The formulated behaviour change intervention bundles (likely 3-5 options) will be presented to a workshop of 7-12 patients and carers. A maximum variation sample of patients from different demographic and socio-economic backgrounds will be recruited.

Workshop 2. The intervention bundles (likely 2-3 refined from workshop 1) will be presented to surgeons, physiotherapists and other healthcare professionals (7-12 in total).

Nominal Group Technique will be used to structure the discussions and prioritise the intervention bundles.

1. *Understand the content and implementation options* using the Template for Intervention Description and Replication (TiDieR) checklist.^17^

The 1-2 intervention bundles prioritised from workshops 1-2 will be explored. Candidate behavioural change techniques (BCTs) derived from the BCT Taxonomy v1 (BCTTv1)^18^ will be selected and incorporated into the intervention bundles.

Workshop 3. All stakeholders from workshops 1 and 2 will be invited to a combined workshop to discuss the selected 1-2 intervention bundles. Using the TiDieR checklist, we will discuss the where, when, how and by whom the interventions could be delivered.

The techniques will be refined to formulate a coherent rehabilitation package. Stakeholders that consent to further contact will be invited to contribute and comment on this process.

Workshops will be conducted face-to-face, virtually via videoconference, or a combination of both. The workshop frequency, duration and format may be amended to cater for participants preferences and availability according to social distancing guidelines in place at the time.

## Discontinuation/Withdrawal of Participants from Study

Each participant has the right to withdraw from the study at any time. All workshops will be audio-record, transcribed and anonymised. If a participant of the workshop meeting withdraws then it may not be possible to withdraw their data as well as it will constitute a part of the flow of workshops and removing it will leave subsequent elements of the discussion out of context.

# ANALYSIS

# Work package 1: Exploratory Analysis

Standard descriptive summaries for patient baseline characteristics and outcome values will be reported. Multiple imputation methods will be used to impute missing data using missing at random assumptions.^19^ To test whether the candidate variables are predictors of outcome, univariate linear regression model analyses for continuous outcomes and logistic regression for binary outcomes will be used. Variables significantly associated with outcomes will be entered into multiple regression models. Three models will be generated with OMAS, EQ5D5L and adverse events as the dependent variable and pre-injury values of the outcome measure and candidate predictors will be entered as independent variables.

# Work package 2: Qualitative Interviews

Qualitative data will be managed using NVIVO 12. Framework analysis will be conducted, deductively coding interview responses into COM-B domains and then inductively generating summary themes from similar responses within domains.^20^ A second researcher will double-code a sub-sample of transcripts to assess COM-B coding reliability. Themes will be discussed and verified during analysis with a multi-disciplinary healthcare group, with data saturation occurring when we agree that no new elements are arising from the data. Findings will be reported using the Consolidated Criteria for Reporting Qualitative Research (COREQ) guidelines.^21^

## Work package 3: Iterative co-design of a rehabilitation package to facilitate safe weight-bearing

Themes from work-package 1 will be mapped onto the COM-B components of the Behaviour Change Wheel, which provides a structured, validated framework for describing behavioural interventions in healthcare.^7^ Where further granularity is required, the 14 domains of the Theoretical Domains Framework will be used to map the themes onto the BCW.^20^

Mapping matrices will be used to identify target interventions functions and Behaviour Change Techniques (BCT’s) that correspond to the behavioural barriers and facilitators to weight-bearing identified from work-package 1. Behavioural intervention bundles will be synthesised and prioritised based on the results of an on-going systematic review of BCT’s in lower limb fracture rehabilitation. These will then be presented to stakeholders and refined at the workshops described in work-package 2, using the APEASE and TiDier criteria.

This will produce a novel, co-designed, flexible rehabilitation package to facilitate safe weight-bearing after ankle fracture surgery.

The content of the rehabilitation package will determine the next steps:

If the outcome is a combination of simple behavioural adjuncts to routine advice, these will be offered as part of the WAX trial dissemination plan. An example might be a short explanation video or a set of prompts to give to healthcare professionals delivering weight-bearing advice.

If the outcome selected is a new therapeutic intervention, further funding will be sought to test this. An example might be an online App or peer support forum.

# QUALITY ASSURANCE PROCEDURES

This research project forms the body of the Lead Investigators DPhil project being undertaken at the University of Oxford. The project will be subject to regular supervision and management review by the named DPhil supervisors in this protocol. In addition, the study may be monitored, or audited in accordance with Good Clinical Practice, relevant regulations and standard operating procedures.

# ETHICAL AND REGULATORY CONSIDERATIONS

National Research ethic Committee approved this study on the 23 March 2021 reference number 19/SC/0566.

# PUBLICATION POLICY

The study report will be prepared and published separately to the WAX trial. Investigators will be involved in reviewing drafts of manuscripts, abstracts, press releases and any other publications arising from the study. Authorship will be determined in accordance with the ICMJE guidelines and other contributors will be acknowledged. No patient identifiable information will be contained in any form of dissemination of study results.

# REFERENCES

1. Collaborative B. Weight-bearing in ankle fractures: An audit of UK practice. Foot Edinb Scotl. 2019 Feb 14;39:28–36.

2. ISRCTN - ISRCTN12883981: Weight-bearing in ankle fractures [Internet]. [cited 2020 Sep 11]. Available from: http://www.isrctn.com/ISRCTN12883981

3. McPhail SM, Dunstan J, Canning J, Haines TP. Life impact of ankle fractures: qualitative analysis of patient and clinician experiences. BMC Musculoskelet Disord. 2012 Nov 21;13:224.

4. Keene D, Mistry D, Nam J, Tutton E, Handley R, Morgan L, et al. The Ankle Injury Management (AIM) trial: a pragmatic, multicentre, equivalence randomised controlled trial and economic evaluation comparing close contact casting with open surgical reduction and internal fixation in the treatment of unstable ankle fractures in patients aged over 60 years. Health Technol Assess Winch Engl. 2016;20(75):1‐158.

5. Craig P, Dieppe P, Macintyre S, Michie S, Nazareth I, Petticrew M, et al. Developing and evaluating complex interventions: the new Medical Research Council guidance. BMJ. 2008 Sep 29;337:a1655.

6. Room J, Hannink E, Dawes H, Barker K. What interventions are used to improve exercise adherence in older people and what behavioural techniques are they based on? A systematic review. BMJ Open. 2017 Dec 1;7(12):e019221.

7. Michie S, van Stralen MM, West R. The behaviour change wheel: a new method for characterising and designing behaviour change interventions. Implement Sci IS. 2011 Apr 23;6:42.

8. Woby SR, Roach NK, Urmston M, Watson PJ. Psychometric properties of the TSK-11: a shortened version of the Tampa Scale for Kinesiophobia. Pain. 2005 Sep;117(1–2):137–44.

9. Bot AGJ, Nota SPFT, Ring D. The creation of an abbreviated version of the PSEQ: the PSEQ-2. Psychosomatics. 2014 Aug;55(4):381–5.

10. Keene DJ, Mistry D, Nam J, Tutton E, Handley R, Morgan L, et al. The Ankle Injury Management (AIM) trial: a pragmatic, multicentre, equivalence randomised controlled trial and economic evaluation comparing close contact casting with open surgical reduction and internal fixation in the treatment of unstable ankle fractures in patients aged over 60 years. Health Technol Assess. 2016 Oct;20:1–158.

11. Lin CW, Donkers NA, Refshauge KM, Beckenkamp PR, Khera K, Moseley AM. Rehabilitation for ankle fractures in adults. Cochrane Database Syst Rev. 2012 Nov 14;11:CD005595.

12. ISRCTN15537280. Ankle injury rehabilitation (AIR). A clinical trial comparing the effectiveness of a plaster cast to a functional brace in the treatment of adults with ankle fractures.

13. Olerud C, Molander H. A scoring scale for symptom evaluation after ankle fracture. Arch Orthop Trauma Surg Arch Orthopadische Unf-Chir. 1984;103(3):190–4.

14. Jayakumar P, Teunis T, Vranceanu A-M, Moore MG, Williams M, Lamb S, et al. Psychosocial factors affecting variation in patient-reported outcomes after elbow fractures. J Shoulder Elbow Surg. 2019 Aug;28(8):1431–40.

15. Jayakumar P, Teunis T, Williams M, Lamb SE, Ring D, Gwilym S. Factors associated with the magnitude of limitations during recovery from a fracture of the proximal humerus: predictors of limitations after proximal humerus fracture. Bone Jt J. 2019;101-B(6):715–23.

16. Michie S. The behaviour change wheel: a guide to designing Interventions. Sutton, Surrey: Silverback Publishing; 2014.

17. Hoffmann TC, Glasziou PP, Boutron I, Milne R, Perera R, Moher D, et al. Better reporting of interventions: template for intervention description and replication (TIDieR) checklist and guide. BMJ. 2014 Mar 7;348:g1687.

18. Michie S, Richardson M, Johnston M, Abraham C, Francis J, Hardeman W, et al. The behavior change technique taxonomy (v1) of 93 hierarchically clustered techniques: building an international consensus for the reporting of behavior change interventions. Ann Behav Med Publ Soc Behav Med. 2013 Aug;46(1):81–95.

19. Sterne JAC, White IR, Carlin JB, Spratt M, Royston P, Kenward MG, et al. Multiple imputation for missing data in epidemiological and clinical research: potential and pitfalls. BMJ. 2009 Jun 29;338:b2393.

20. Atkins L, Francis J, Islam R, O’Connor D, Patey A, Ivers N, et al. A guide to using the Theoretical Domains Framework of behaviour change to investigate implementation problems. Implement Sci IS. 2017 21;12(1):77.

21. Tong A, Sainsbury P, Craig J. Consolidated criteria for reporting qualitative research (COREQ): a 32-item checklist for interviews and focus groups. Int J Qual Health Care J Int Soc Qual Health Care. 2007 Dec;19(6):349–57.
